# Independent Evolution of Linear and Branched Cuticular Hydrocarbons in Ants: A Hypothesis

**DOI:** 10.3390/insects17040427

**Published:** 2026-04-16

**Authors:** Abraham Hefetz

**Affiliations:** School of Zoology, Faculty of Life Sciences, Tel Aviv University, Tel Aviv 6997801, Israel; hefetz@tauex.tau.ac.il

**Keywords:** *Cataglyphis*, cuticular hydrocarbons, branched fatty acids, branched alkane evolution

## Abstract

The epicuticle of *Cataglyphis niger* is endowed with hydrocarbons comprising both linear and branched alkanes. For linear alkane, it is hypothesized that the primary driving force for their evolution was acquiring means for attaining cuticular impermeability that protects the ants from desiccation, and, secondarily, was co-opted as cues and signals. For example, being more abundant in foragers, they signal colony foraging intensity and, accordingly, adjust task allocation. Branched alkanes serve mostly in communication, but their evolution is less clear. Studies of the biosynthesis of both classes of hydrocarbons revealed disparate pathways, which suggests an independent evolution. The biosynthesis of branched alkanes hints at their possible evolution. They are derived from branched fatty acids, which have evolved as protective means due to their bactericidal activity. It is hypothesized that their biosynthetic pathway was secondarily co-opted for producing branched alkanes as signals and cues. Branched alkanes blend within the linear alkane layer to evenly cover the ants’ body surface and enhance the ants’ communicative capacity by conveying larger informational content due to their numerous positional and stereoisomers. The present study presents the occurrence of substantial amounts of branched fatty acids with branching position that matches that of the branched alkanes. The disparate biosynthesis pathways and the postulated differences in their evolutionary roots lend credence to the hypothesis of their independent evolution.

## Simple Summary

The surface of the ant’s body is covered with a thin layer of hydrocarbons consisting of linear and branched alkanes. For linear alkanes, the prevailing hypothesis is that they evolved primarily to form a cuticular impermeable layer, without which the ants rapidly desiccate and die. Subsequently, they may have been co-opted as communicative cues and signals. For branched alkanes, behavioral evidence indicates that they mainly function in communication, but their evolution is less clear. Their occurrence on the cuticular surface seems to be a tradeoff between two features: on the one hand, they are maladaptive because they reduce cuticular impermeability, while on the other hand, they are adaptive as they encode more information through multiple branching positions and stereoisomers. The fact that linear and branched alkanes are biosynthesized via distinctly clear and non-overlapping pathways suggests their independent evolution. Linear alkanes are biosynthesized from acetate and mevalonate with linear fatty acids as intermediates. Branched alkanes, on the other hand, are biosynthesized from amino acids with branched fatty acids as intermediates, which hints at their possible evolution. Branched fatty acids are known bactericides, in particular against mycoplasma that lack cell walls, which may have been the driving force for their evolution. Here, I hypothesize that branched fatty acids evolved first as bactericides and subsequently branched alkanes evolved by co-opting the already established biosynthetic pathway to serve as cues and signals. Moreover, being hydrophobic, they readily blend chemically with linear hydrocarbons, which enabled their smooth and even distribution on the cuticular surface. Here, I present evidence for the occurrence of branched fatty acids in head extracts of the desert ant *Cataglyphis niger*, the branching position of which matched that of the branched hydrocarbons. These acids are accompanied by corresponding heptyl esters, which presumably act as a non-toxic storage form. The acids and their esters are absent from the major cephalic exocrine gland, the postpharyngeal gland, indicating that they originate from another yet unidentified gland. The occurrence of the branched fatty acids in substantial, rather than catalytic, amounts, and the congruency of their branching positions with that of the branched alkanes lend credence to the above hypothesis of branched alkanes evolution.

## 1. Introduction

**Figure 1:**
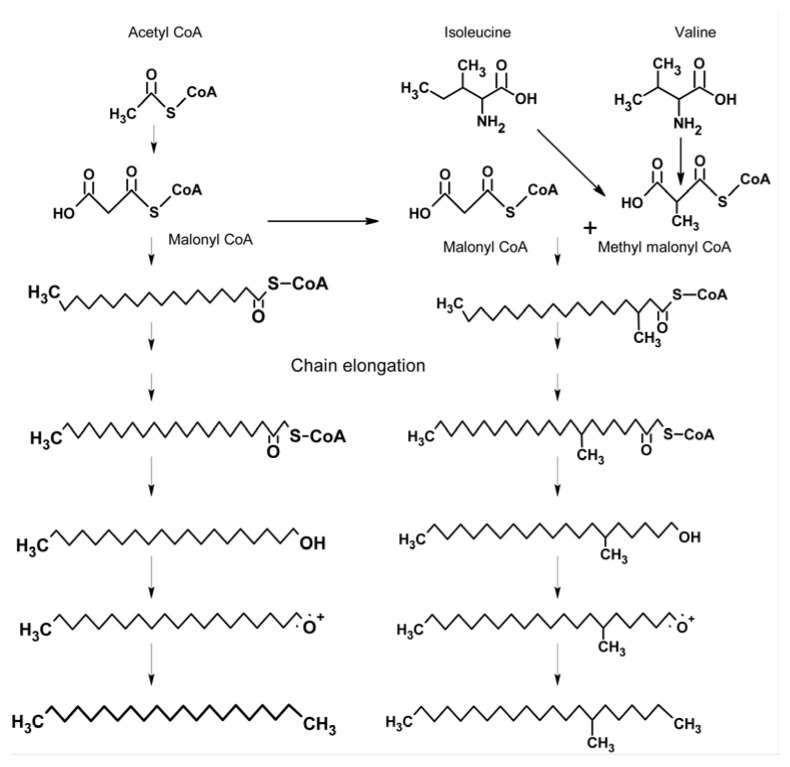
The biosynthesis of linear (**left**) and branched (**right**) alkanes. Adapted from [11,14].

The insect epicuticle is endowed with a plethora of hydrocarbons (cuticular hydrocarbons—CHCs) comprising saturated and unsaturated linear alkanes, and multiple monomethyl and dimethyl-branched alkanes. They encompass dual function: to provide an impermeable layer that protects the insect from desiccation, as reviewed in [1], and in communication, as reviewed in [2]. To achieve impermeability, the epicuticle comprises multiple linear alkanes, generally ranging from eicosane (C_20_H_42_—molecular weight MW 282) to tritriacontane (C_33_H_68_—MW 464), that cover the entire ant’s cuticular surface. The use of a mixture of alkanes, rather than a single high-molecular alkane, is adaptive because the mixture expresses a eutectic behavior; that is, the mixture behaves as a single compound with a melting point that is below any of its pure components, which creates a uniform solid–liquid layer that overlays the entire body surface. Impermeability increases with the increase in carbon chain length, so the mixture composition seems to be a tradeoff between the gain in impermeability and the cost of biosynthesis. In social insects, ants in particular, the CHC is typified by the occurrence of multiple branched alkanes, mostly monomethyl but also dimethyl compounds. This is perplexing because the presence of branched alkanes lowers the melting temperature of the linear alkane mixture and consequently lessens the epicuticle efficacy as an impermeable layer [3,4]. This suggests that in ants, the branched CHCs have another essential function. Behavioral studies have indeed shown that the branched CHCs are effective mediators of social communication, mostly regarding nestmate recognition but also in delineating caste identity, e.g., queen recognition [2,5,6,7,8,9]. The use of branched alkanes for communication is adaptive because they potentially contain an abundance of chain-position isomers (within-molecule variation in the branching sites) as well as potential stereoisomers, which greatly increases their information content as chemical signals [2,10,11,12,13]. Ant-branched CHCs are generally non-volatile, so their mode of perception is mostly through contact that, in turn, necessitates their externalization onto the body surface. They therefore constitute a blend with the linear alkanes, conferring a dual function for the CHCs in total. Intuitively, the evolution of branched alkanes as cuticular components may have been derived from the biosynthesis of the respective linear alkanes by adding the branching. But, in fact, studies of the biosynthesis of branched alkanes have shown that they are biosynthesized via a specific pathway, disparate from the pathway of linear alkanes (Figure 1; [14]).

In view of their distinct biosynthetic pathways, I propose a hypothesis of an independent evolution of linear and branched alkanes. Considering the notion that linear alkanes evolved primarily to impart impermeability and, secondarily, as cues and signals, what was the root for the evolution of branched alkanes? Did they primarily evolve in response to communication needs or originally fulfilled a different function and were secondarily adopted as communicative signals? From the parsimony perspective of evolution, the secondary adoption of branched alkanes for communication seems a most probable hypothesis. If so, what was their postulated non-communicative function? I further propose the hypothesis that branched fatty acids, which are precursors of branched alkane biosynthesis, may have been the root of their evolution. Branched fatty acids are effective bactericides that disrupt the bacterial cell membrane, and are, in particular, effective against mycoplasma that lack a cell wall [15]. Mycoplasmas are abundant in arthropods and include entomopathogenic species [16], which suggests that this was the original driving force for the evolution of branched fatty acids. Thereafter, their biosynthetic pathway was co-opted to generate branched alkanes as communicative signals, which, due to their hydrophobicity, blended well with the linear alkanes and readily spread throughout the body surface. The following hypothesis posits that if branched fatty acids act as bactericides, ants must still produce them in substantial rather than catalytic amounts. The present study shows, by chemical analysis of head extracts in the ant *Cataglyphis niger*, the presence of multiple branched fatty acids, with branched positions that correspond to the ants’ cuticular branched alkanes. Furthermore, it appears that they are stored in the head as heptyl esters, presumably for protecting the glandular cells from the ill effects of free branched fatty acids.

## 2. Materials and Methods

### 2.1. Ant Collection

Colonies of *Cataglyphis niger* (n = 3) were collected in Tel-Aviv, Israel, and included the brood, queens, and workers. In the laboratory, the ants were transferred to artificial nests placed in a rearing room under a controlled temperature of 28 ± 2 °C and a photoperiod of 14L:10D. The ants were provided with a diet of sugar water and minced insects three times a week.

### 2.2. Chemical Analysis

All extracts and dissections were performed using freshly frozen workers. For total head extracts, heads from 10 workers were cleanly excised, pooled and homogenized in 1 mL of hexane. The extracts were filtered through a sinter glass funnel to remove all cuticular debris and concentrated to approximately 200 µL. Postpharyngeal glands were cleanly dissected out of the head by removing the labium and pinching out the gland with fine forceps. For extraction, each gland (n = 3) was placed in 200 µL of hexane. All extracts were stored at −20 °C until analysis.

Chemical analyses were performed by combined gas chromatography mass spectrometry, Agilent 19091S-433UI (Agilent, Tel Aviv, Israel) at EI (70 ev), helium carrier gas at a flow of 24.2 mL/min, an injector temperature of 250 °C and splitless modes. Compound separation was achieved using a HP-5 ms column that was temperature programmed from an initial temperature of 60 °C with a 1 min hold, followed by a temperature rise at a rate of 10 °C/min to 300 °C, and a final hold of 15 min. Compounds were identified by their mass fragmentation pattern. Retention times were ascertained by injecting a series of alkanes from decane (C_10_H_12_) to tritriacontane (C_33_H_68_) under the same conditions.

## 3. Results

**Figure 2:**
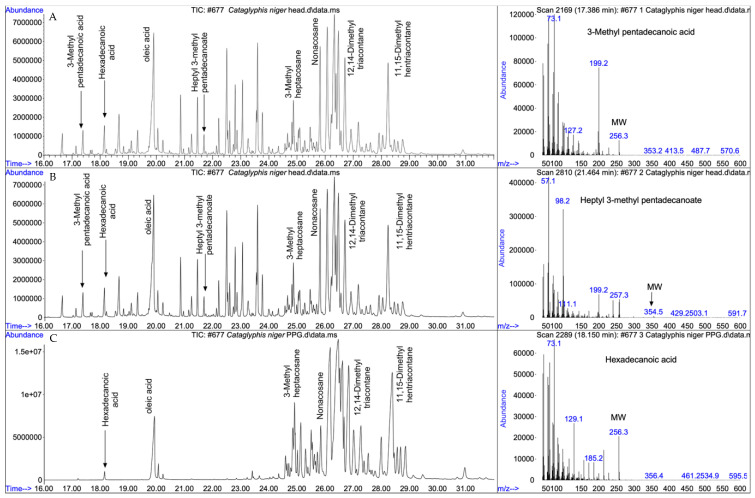
Total ion chromatograms of extracts of various body parts of *Cataglyphis niger*: (**A**) head extract and the mass spectrum of 3-methyl pentadecanoic acid; (**B**) head extract and the mass spectrum of heptyl 3-methyl pentadecanoic acid; (**C**) cleanly dissected postpharyngeal gland and the mass spectrum of hexadecanoic acid.

Figure 2 shows a qualitative comparative analysis of head extracts and a cleanly dissected postpharyngeal gland, along with three spectra characterizing the three types of compounds identified. Table 1 shows the fatty acid and their corresponding heptyl ester components of the total head extracts, and Table 2 shows the alkane components of the postpharyngeal gland secretion. Head extracts possess three classes of compounds: linear and branched fatty acids (C_14_–C_18_), their corresponding heptyl esters, and aliphatic hydrocarbons. The identity of the branched fatty acids was inferred from their mass spectra. For comparison, linear hexadecanoic acid (molecular weight 256; Figure 2C, right) elutes at 18.1 min and its spectrum is typified by a large molecular ion (m/z 256) and large fragments at m/z 60 and m/z 73 (corresponding to acetate and a higher rearrangement at the carboxylic end of the molecule). On the other hand, 3-methyl pentadecanoic acid (Figure 2A, right) has the same molecular weight of 256 but elutes earlier, at 17.4 min. Its spectrum is characterized by a much smaller molecular ion (m/z 256) and a large ion at m/z 199, indicating the loss of 57 (C_4_H_9_). This compound also has an appreciably lower ion at m/z 60, compared to hexadecanoic acid. The monomethyl fatty acids were occasionally accompanied by dimethyl fatty acids, deduced from their molecular ion and the lower retention time (RT) compared to the same molecular weight linear or monomethyl fatty acids. For example, for a molecular weight of 270, the RT for dimethyl pentadecanoic acid was 18.2 min, for 3-methyl hexadecanoic acid, an RT of 18.6 min, and for heptadecanoic acid, an RT of 18.8 min. The small quantities of the dimethyl acids, however, did not allow full determination of the branching positions. The omnipresent loss of 57 from the molecular ion (m-57) indicates a branching at the 3-position, but the determination of the second branching position was not possible.

The second type of compounds found in head extracts are heptyl esters, as judged by the large ion peak at m/z 98, corresponding to the heptane fragment of the alcoholic moiety of the ester. As in the case of the branched carboxylic acids, the identity of the acidic moiety was characterized by its corresponding molecular ion and (m-57) for the 3-methyl acids. The molecular weight of each of the esters was disclosed by a small but visible molecular ion. For example, the ester heptyl 3-methyl pentadecanoate (Figure 2B, right) is characterized by the molecular ion at m/z = 354, with m/z = 98 corresponding to of heptane representing the alcoholic moiety of the ester, m/z = 257 indicating the acid moiety of the ester, and m/z 239 and m/z 199 corresponding, respectively, to the loss of water and butane from the acid moiety.

The third type of compounds comprised linear and branched alkane ranging from pentacosane to tritriacontane, which were common both in the head extracts and the postpharyngeal gland, indicating that the latter is their source. Notable is the dominance of the high-molecular-weight hydrocarbons ranging from mono- and dimethyl nonacosane (MW 422–436) to mono- and dimethyl tritriacontane (MW 478–492), in line with the desertic habitat these ants occupy. The postpharyngeal gland exudates also contained hexadecanoic, oleic, and octadecanoic acids, but none of the branched acids or their esters, indicating that the origin of both the branched acids and their corresponding esters is from another, yet unknown head gland.

## 4. Conclusions

The evolution of communicative signals is complicated by the requirement that its two components, the signal and its perception, have to coevolve to be effective. The general assumption is of a two-step evolution. For chemical communication, for example, it is assumed that preexisting substances that fulfilled a structural (non-communicative) function were secondarily co-opted as communication signals. The evolution of cuticular hydrocarbons serves as an example of such an evolutionary scenario. They constitute mostly two classes, linear alkanes and monomethyl and, to a lesser extent, dimethyl-branched alkanes. Multiple studies have shown that the linear alkane components are important in imparting an external impermeable layer that protects the ants from desiccation ([1] and references therein). Covering the external surface of the body made them ideal for co-option as communicative cues and signals. Moreover, theoretically, only a few long-chain linear alkanes are sufficient for imparting an effective impermeable layer. Yet, as exemplified in this study, cuticular hydrocarbons comprise a complex mixture of linear alkanes. This suggests that they bear another function, namely communicative function. A presumed subsequent evolution of specific hydrocarbon perception systems [17,18,19,20] paved the way for their co-option as signals for nestmate recognition as well as denoting caste identity [2,7,21,22]. The cooccurrence of branched alkanes that may contain even larger bouts of information added another dimension to the role of cuticular hydrocarbons in communication.

While the role of hydrocarbons in providing an impermeable layer is not necessarily idiosyncratic, their co-option for communication needs to be species and even caste-specific, which led to diversification in their composition along the phylogeny of ants [23]. In that regard, branched alkanes are great substrates for diversification because, for a given chain length, each of the linear alkanes can possess multiple positional isomers (the molecular site of the branching), enhancing the informational content of the semiochemical. A caveat in utilizing an abundance of branched alkanes is that this diminishes the impermeability efficacy of the cuticle. Therefore, there seems to be a flexible steady state with regard to the proportion of each of these classes of hydrocarbons.

While the hypothesis that the linear alkanes’ role as communicative cues and signals evolved from their role in imparting impermeability is generally accepted, the question of how branched alkanes evolved remains unclear. Did branched alkanes derive from linear alkanes, or did they evolve independently? The fact that these two classes of alkanes are biosynthesized via separate pathways lends credence to their independent evolution, rather than as derivatives of linear alkanes. What was then the driving force for their evolution? Did they evolve specifically as signals, or have they evolved in response to another, non-communicative, function and were secondarily co-opted as signals? Inspection of the biosynthesis of branched alkanes points to branched fatty acids as roots of their evolution. Branched fatty acids, in particular the 3-methyl fatty acids, are effective bactericides and may have evolved to protect the ants from microbial pathogens such as mycoplasma. It was but a small biosynthetic leap to generate the corresponding branched alkanes [14,24]. Corroborating this hypothesis are the above-described discovery of multiple branched fatty acids in *C. niger* head extracts in larger amounts than expected from being biosynthetic intermediates. Their absence from the postpharyngeal gland content, the major storage of cuticular hydrocarbons (both linear and branched), indicates that they are stored elsewhere in the head. Although not specifically tested in ants, the possibility that they have retained their function as bactericides and are applied to the body surface by extensive self-grooming typical of these ants is a sensible hypothesis [25]. The head extracts also contained large amounts of their corresponding heptyl esters, probably serving as a benign storage of the rather toxic branched fatty acids.

The above hypothesis may explain the occurrence of anteiso-branched alkanes, but not the extensive branching in the middle of the alkane chain. This is explained by the need for signal diversification, achieved by generating branched positional isomers. Biosynthetically, this is made possible by the combinatorial coupling of mevalonate and methyl mevalonate intermediates. Moving the branching position, however, created another problem because, in contrast to the 3-methyl alkanes that do not reduce much the cuticular impermeability, middle-branched alkanes considerably decrease it [26]. What then might be their advantage that counterbalances their disadvantage? One hypothesis may be that these compounds could constitute costly signals, sensu the handicap principle [27,28], encoding yet undescribed communicative function, e.g., queen–worker signaling. Accordingly, the 3- and 5-methyl-branched fatty acids constitute the protective zone, whereas the middle 11- and 13-methyl-branched fatty acids constitute the handicap zone (illustrated in Figure 4.4 in [11]).

Reinforcing the hypothesis of the independent evolution of linear and branched alkanes is a recent study of the regulation of their biosynthesis in the Carpenter ant *Camponotus fellah*. It is regulated by the neurohormone inotocin produced by the suboesophageal ganglion that is perceived in the fat body by a specific inotocin receptor. Expressions of both the neurohormone and its receptors are upregulated when workers shift from nursing to foraging, resulting in a concomitant increase in the proportion of linear alkanes. Blocking the inotocin receptor with the inhibitor Atociban resulted in a significant reduction in the amounts of linear alkanes but not that of branched alkanes [29]. This suggests a different effect of inotocin on the biosynthesis of linear vs. branched alkanes, reaffirming their independent evolution. Notwithstanding, using another inotocin antagonist (designated as compound B in [29]) resulted in a decrease in both linear and branched alkanes, possibly affecting their biosynthesis in a different way.

In conclusion, there are several hypotheses pertaining to the evolution of linear and branched hydrocarbons as communicative cues and signals. The first hypothesis that linear and branched alkanes evolved independently stems from their disparate biosynthetic pathways and differential neurohormonal regulation. A second hypothesis posits that linear alkanes evolved primarily to impart impermeability and secondarily for communication. In ants, their levels are regulated in accordance with the shift from indoor to outdoor tasks. Being higher in foragers, they may also act as a cue that indicates the foraging performance of the colony and, consequently, affect overall colony behavior [30]. The third hypothesis posits that branched alkanes may have evolved by co-opting the preexisting biosynthetic pathway of the antibiotic branched fatty acid and utilizing them for communication, e.g., nestmate recognition and caste idiosyncrasy. Assuming a communicative role, the necessity for more specificity ensued and was a driving force for their molecular diversification, including the shift in branching towards the middle of the molecule. The amount of branching and the abundance of middle-chain branching may be regulated as a tradeoff between lowering impermeability and reliably but costly signaling. Once the tradeoff has stabilized, the composition of branched alkanes is independent of task allocation and thus does not practically change with age or task.

## Figures and Tables

**Table 1:** The acid and their heptyl ester constituents of the total head extracts of *Cataglyphis niger*.

RT	Compound
16.656	3-methyl tetradecanoic acid
17.143	3,x-dimethyl tetradecanoic acid
17.390	3-methyl pentadecanoic acid
17.711	3,x-dimethyl pentadecanoic acid
18.154	hexadecanoic acid
18.226	11-methyl hexadecanoic acid
18.549	5-methyl hexadecanoic acid
18.673	3-methyl hexadecanoic acid
18.835	heptadecanoic acid
19.030	11-methyl heptadecanoic acid
19.111	5-methyl heptadecanoic acid
19.334	3-methyl heptadecanoic acid
19.906	oleic acid
20.863	heptyl 3-methyl tetradecanoate
20.053	stearic acid
21.153	heptyl 3,x-dimethyl tetradecanoate
21.252	heptyl pentadecanoate
21.464	heptyl 3-methyl pentadecanoate
21.694	heptyl 3,x-dimethyl pentadecanoate
22.221	heptyl hexadecanoate
22.506	heptyl 5-methyl hexadecanoate
22.610	heptyl 3-methyl hexadecanoate
22.802	heptyl heptadecanoic acid
23.065	heptyl 3-methyl heptadecanoate
23.599	heptyl oleate
23.772	heptyl stearate
24.001	heptyl 3-methyl octadecanoate
24.129	heptyl nonadecanoate

**Table 2:** The hydrocarbon constituents of total head extracts and dissected postpharyngeal gland of *Cataglyphis niger*.

RT	Compound
22.848	pentacosane
23.228	5-methyl pentacosane
23.412	3-methyl pentacosane
23.456	5,9-dimethyl pentacosane
23.615	hexacosane
23.662	3,9-dimethyl pentacosane
24.036	4-methyl hexacosane
24.152	2-methyl hexacosane
24.351	heptacosane
24.593	11- + 13-methyl heptacosane
24.645	7-methyl heptacosane
24.711	5-methyl heptacosane
24.781	11,15-dimethyl heptacosane
24.855	7,11-dimethyl heptacosane
24.917	3-methyl heptacosane
25.017	11,13,15-trimethyl heptacosane
25.136	9,13,15-trimethyl heptacosane
25.306	12-methyl octacosane
25.388	6-methyl octacosane
25.507	4-methyl octacosane
25.545	8,12-dimethyl octacosane
25.618	6,12-dimethyl octacosane
25.662	nonacosene
25.728	4,12-dimethyl octacosane
25.847	nonacosane
26.181	9- + 11- + 13- + 15- methyl nonacosane
26.468	9,13 dimethyl nonacosane
26.525	7,11- + 7,13-dimethyl nonacosane
26.622	5,11- + 5,13-dimethyl nonacosane
26.676	7,11,15-trimethylthyl nonacosane
26.840	3,11- + 3,13 dimethyl nonacosane
27.017	12- + 14- methyl triacontane
27.119	6-methylthyl triacontane
27.274	10,14-dimethyl triacontane
27.387	6,14-dimethyl triacontane
27.536	4,12-dimethyl triacontane
27.670	hentriacontane
27.999	11-methyl hentriacontane
28.390	11,15-dimethyl hentriacontane
28.457	7,15-dimethyl hentriacontane
28.567	5,15-dimethyl hentriacontane
28.687	7,11,15-trimethyl hentriacontane
28.861	5,11,15-trimethyl hentriacontane
29.138	14-methyl dotriacontane
29.472	12,16-dimethyl dotriacontane
30.699	11-methyl tritriacontane
30.972	11,15-dimethyl tritriacontane
31.497	7,11-dimethyl tritriacontane
31.721	5,11-dimethyl tritriacontane

## Data Availability

The original contributions presented in this study are included in the article. Further inquiries can be directed to the corresponding author.
